# The high-risk B-to-E transitional phenotype: a prospective multi-centre validation of patient reclassification under the updated GOLD 2026 criteria

**DOI:** 10.7189/jogh.16.04205

**Published:** 2026-07-17

**Authors:** Boyan Zhang, Junhao Zeng, Huihui Zeng, Zhongshang Dai, Yan Chen

**Affiliations:** 1 Department of Respiratory and Critical Care Medicine, The Second Xiangya Hospital of Central South University, Changsha, China; 2 National Clinical Research Centre for Endocrine and Metabolic Diseases and Department of Metabolism and Endocrinology, The Second Xiangya Hospital of Central South University, Changsha, China; 3 Department of Infectious Diseases, The Second Xiangya Hospital, Central South University, Changsha, China

**Keywords:** chronic obstructive pulmonary disease, Global Initiative for Chronic Obstructive Lung Disease, risk stratification, acute exacerbation, prospective cohort

## Abstract

**Background:**

The Global Initiative for Chronic Obstructive Lung Disease (GOLD) 2026 criteria lowered the threshold for classifying Group E to ≥1 moderate or severe acute exacerbation in the last year. However, the scale of patient reclassification and its clinical justification in real-world settings have not been validated.

**Methods:**

We utilised a prospective, multi-centre Chinese chronic obstructive pulmonary disease (COPD) cohort (n = 966), and applied the GOLD 2015, 2025, and 2026 criteria to the same cohort, focusing on patients who shifted from group B under GOLD 2025 to group E under GOLD 2026. The primary outcome was time to first moderate-to-severe acute exacerbation; secondary outcomes included symptom burden, health care resource utilisation, and clinical features of patients in the subgroups.

**Results:**

Of 952 analysable participants, 242 were classified as shift-to-E. During an average follow-up of 10.3 months, the shift-to-E subgroup showed a 92% higher risk than stable B (hazard ratio (HR) = 1.92; 95% confidence interval (CI) = 1.14–3.23, *P* = 0.01441). Its adjusted follow-up scores of the COPD assessment test (CAT = 14.83) and the modified Medical Research Council dyspnoea scale (mMRC = 2.191) were comparable to stable E (CAT = 15.30 and mMRC = 2.214) and significantly higher than stable B. The subgroup also had longer baseline hospital stays (12.31 days) than stable B (8.246 days), supporting the utility of the GOLD 2026 for precise COPD stratification.

**Conclusions:**

The GOLD 2026 update effectively identifies a substantial subgroup of patients with a distinct high-risk B-E transitional phenotype. Their exacerbation risk and symptom burden approximate those of the traditional high-risk group E, providing direct prognostic evidence for treatment escalation based on a history of just one moderate exacerbation. This supports the clinical utility of the new criteria for precise risk stratification.

**Registration:**

Chinese Clinical Trial Registry (ChiCTR-POC-17010431).

Chronic obstructive pulmonary disease (COPD), as one of the leading global causes of disability and mortality, has its core of disease management lying in accurate risk assessment and stratified treatment [1]. Since its inaugural publication in 2001, the Global Initiative for Chronic Obstructive Lung Disease (GOLD) criteria have established themselves as the global benchmark for the diagnosis, assessment, and management of COPD [2,3].

Several crucial updates have been made to clarify the pathophysiology and recommended treatment within the framework of evidence-based medicine, particularly the comprehensive assessment and classification of disease severity. The four-group (A, B, C, and D) classification was first established in GOLD 2011, and the 2015 GOLD update marked an essential shift in the assessment paradigm: it abandoned lung function as a criterion for classification and incorporated both patients’ symptom burden and a history of acute exacerbations, aiming to better identify individuals with mild symptoms but high future risk, as well as those with significant symptoms but relatively low risk, thereby promoting individualised treatment decisions [4,5]. Subsequently, based on the growing consensus that frequent acute exacerbations serve as a core adverse prognostic indicator, GOLD 2023 further simplified the classification by merging groups C and D, previously defined by symptom-risk combinations in the older version, into a unified group E, explicitly defined as patients experiencing ≥2 moderate acute exacerbations or ≥1 severe exacerbation requiring hospitalisation per year. This revision strengthens the management strategy centred on the exacerbator phenotype, aiming to ensure that high-risk populations receive more proactive interventions [6]. Such classification criteria were continued in GOLD 2024 and GOLD 2025 [7].

Recently, GOLD 2026 has further lowered the threshold for defining group E to ≥1 moderate or severe acute exacerbation in the preceding year [8,9]. The theoretical basis for this revision lies in the growing evidence that even a single moderate exacerbation is a strong predictor of future recurrent exacerbations, accelerated lung function decline, increased hospitalisation rates, and elevated mortality risk [10–12]. Therefore, lowering the threshold for therapeutic intervention aims to achieve earlier risk identification and prevention, halting the disease from entering a vicious cycle of frequent exacerbations.

However, the real-world clinical implications of this revision remain unclear. Key questions include the extent of patient reclassification and whether newly identified group E patients, particularly those shifting from group B, share clinical profiles and prognoses with traditional high-risk Group E patients, thereby justifying their reclassification. Currently, robust prospective evidence addressing these questions is lacking.

Therefore, we aimed to evaluate the impact of the GOLD 2026 update using a prospective multi-centre COPD cohort. To place GOLD 2026 in the context of our research, we classified the same cohort under three successive GOLD frameworks. As we introduced above, we considered GOLD 2015 the earlier ‘A-B-C-D’ paradigm. GOLD 2025 represents the immediately preceding ‘A-B-E’ framework with relatively strict criteria, and GOLD 2026, as the newest GOLD guideline, introduces a lower threshold for acute exacerbations in the prior year. This stepwise comparison allows direct quantification of the incremental reclassification attributable specifically to the 2026 revision. We specifically assessed reclassification patterns, focusing on patients who shifted from group B to group E (‘shift-to-E’ subgroup), the subgroup’s multidimensional characteristics, and its short- to medium-term outcomes compared with stable group B and E patients.

## METHODS

### Study population and cohort information

**Figure 1:**
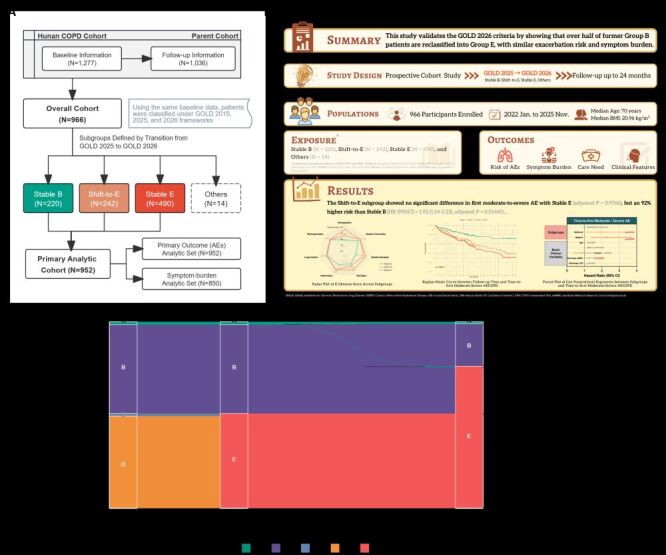
Summary of this study.Panel A. Cohort flowchart. Panel B. Graphical abstract. Panel C. Sankey plot of patient group reclassification across GOLD 2015, 2025, and 2026 criteria.

The Hunan COPD cohort (total n = 1,277 and follow-up n = 1,036), the parent cohort of our study, has been described in our prior publication, in which the inclusion and exclusion criteria were also detailed [13]. This study is a retrospective analysis of a prospective cohort, using data collected between January 2022 and November 2025, comprising 966 participants (the ‘overall cohort’) (**Figure 1**, Panel A).

Ethics Committee of the Second Xiangya Hospital of Central South University (approval number 2016076fabh001) approved the study, which was also registered in the Chinese Clinical Trial Registry (registration number ChiCTR-POC-17010431), and conducted in accordance with the Declaration of Helsinki. Written informed consent was obtained from all participants. We conducted and reported a study in accordance with the STROBE guidelines (Table S1 in the **Online Supplementary Document**).

Regular follow-up was conducted via telephone or face-to-face interviews at one, three, six, 12, and 24 months post-discharge, and during any unplanned re-admissions for treatment. During each follow-up visit, a detailed assessment was performed of patients’ clinical symptoms, history of acute exacerbations, exposure to risk factors, and medication adherence and usage. All information was collected using standardised case report forms (CRFs) and was based on patient self-report, with verification from electronic medical records where available. The same assessment protocols and CRFs were applied uniformly across all 12 participating tertiary hospitals to ensure data consistency.

### Exposure and grouping methods

In this study, we primarily focused on the innovative grouping approach of GOLD 2026, which is adopted as the core exposure factor. Patients were stratified once using the same baseline information and acute-exacerbation history, then remapped to the GOLD 2015, 2025, and 2026 frameworks to better quantify changes in classification across guideline versions. We counted the number of acute exacerbations in the year before patients’ admission and used to group patients, along with their baseline modified Medical Research Council dyspnoea scale (mMRC) and COPD assessment test (CAT) scores, evaluated at admission. We defined a moderate exacerbation as an acute worsening of respiratory symptoms requiring treatment with systemic corticosteroids and/or antibiotics, whereas a severe exacerbation was defined as an exacerbation requiring hospitalisation. Therefore, we categorised all patients in the ‘overall cohort’ into four subgroups reflecting their transition from the GOLD 2025 to the 2026 classification: stable B (n = 220, group B in both years), shift-to-E (n = 242, group B in 2025 reclassified as E in 2026), stable E (n = 490, group E in both years), and others (n = 14, including group A patients and those with missing data). No repeated reclassification based on new clinical measurements was performed. For subgroup comparisons, patients in the stable B, shift-to-E, and stable E groups constituted the ‘primary analytic cohort’ (n = 952), whereas the ‘others’ subgroup was excluded from inferential analyses due to its relatively small size, heterogeneous characteristics, and our research goal of focusing on the GOLD 2026 criteria change of ‘E subgroup.’

### Outcomes

We evaluated the following four primary outcome categories. Acute exacerbation risk in the follow-up period – the primary outcome was the time from discharge to the first moderate or severe acute exacerbation. A secondary outcome was the proportion of patients who experienced ≥1 severe exacerbation during follow-up. Symptom burden – the measures encompassed the alteration in the modified mMRC dyspnoea scale score and the CAT score at discharge, in addition to the follow-up CAT score, mMRC score, and their respective changes from discharge. The mMRC ranges from zero to four, with higher scores indicating more severe dyspnoea. The CAT ranges from zero to 40, with higher scores indicating worse COPD-related health status. In GOLD-based symptom assessment, a CAT score ≥10 or an mMRC score ≥2 is commonly used to indicate a higher symptom burden. Care need – whether home oxygen therapy or home non-invasive positive pressure ventilation (NIPPV) was required, and the reported duration of daily use (hours) was assessed based on patients’ reports and the electronic medical record during the follow-up period. Oxygen flow rate was not available in the present analytic data set and was therefore not analysed. Clinical features – baseline hospital length of stay and laboratory indices, including arterial partial pressure of oxygen, oxygen saturation, lung function indexes forced expiratory volume in one second (FEV_1_), first second forced expiratory volume as a percentage of predicted (FEV_1_%pred), FEV_1_ after bronchodilator, FEV_1_%pred after bronchodilator, FEV_1_/forced vital capacity (FVC), FEV_1_/forced vital capacity percentage predicted (FVC%pred), FEV_1_/FVC after bronchodilator, FEV_1_/FVC%pred after bronchodilator), C-reactive protein (CRP), procalcitonin, white blood cell count, neutrophil count, and eosinophil count were compared among subgroups.

### Statistical analysis

#### Descriptive statistics

We presented continuous variables conforming to a normal distribution as means (x̄) and standard deviation and compared using analysis of variance. Non-normally distributed variables are presented as median (interquartile range) and compared using the Kruskal-Wallis test. Categorical variables are presented as frequencies (percentages) and compared using the χ^2^ or Fisher exact test, as appropriate.

#### Domain-based supervised similarity scoring

We divided the baseline information into seven subdomains based on clinical significance (Table S1 in the **Online Supplementary Document**). The above variables were collected at the baseline assessment around the admission date and were modelled and scored independently within each field. Stable B and stable E subgroups were utilised to train under a generalised linear model with a binomial distribution and a logit link function. An E-likeness score was computed as the outcome label, with stable B assigned zero and stable E assigned one. Then we entered the shift-to-E subgroup data, calculated its E-likeness score, and printed it to determine whether its clinical features are closer to the stable B or stable E subgroup.

#### Survival analysis and pairwise comparison

For the time from discharge to the first moderate-to-severe acute exacerbation, we plotted Kaplan-Meier curves and used the log-rank test to compare groups. When a significant overall difference was observed, we performed pairwise comparisons between all three subgroups with Bonferroni adjustment, and the adjusted *P*-value was reported. For the primary survival analysis, only the first moderate-to-severe exacerbation during follow-up was considered, and subsequent events were excluded.

We summarised recurrent events separately as follow-up exacerbation counts and in the binary endpoint of ≥1 severe exacerbation during follow-up. We used logistic regression to compare the binary endpoint across different subgroups.

#### Cox proportional regression analysis

We used univariable and multivariable Cox proportional hazards regression models to estimate hazard ratios (HRs) with 95% confidence intervals (CIs) for the first moderate-to-severe exacerbation across subgroups (with the stable B subgroup as the reference). We adjusted the multivariable model for age, sex, discharge mMRC score, and discharge CAT score. Additionally, for continuous outcomes such as symptom scores and treatment duration, we employed the Wilcoxon rank-sum test, analysis of covariance, or Gamma regression models to compare groups. The proportional hazard (PH) test and Schoenfeld’s residual error plot were used to assess whether a time-dependent trend exists.

As a sensitivity analysis, we conducted a Firth penalised Cox proportional hazards regression to evaluate the relationship between the results and the modelling methods. Analyses were performed on an endpoint-specific complete-case basis. Participants missing variables required for a given outcome were excluded from that specific analysis, and no imputation was performed. We conducted all analyses with *R*, version.4.5.1 (R Core Team, Vienna, Austria), and a two-sided *P*-value <0.05 was considered statistically significant.

## RESULTS

### Baseline characteristics

A total of 966 patients from the Hunan COPD cohort were included in this study (**Figure 1**, Panel B). The baseline characteristics indicated a gradient relationship in the shift-to-E subgroup compared with the stable B and stable E subgroups, including lung function indices (*e.g.* FEV_1_%pred, FEV_1_/FVC), CRP levels, and baseline CAT scores (Table S2 in the **Online Supplementary Document**). Among subgroups, we identified a bias toward males (92.5%) and an older age profile (mostly elderly patients). Disease duration also differed across subgroups, with the stable E subgroup showing the longest time since first COPD diagnosis, the stable B subgroup the shortest, and the shift-to-E subgroup occupying an intermediate position.

We performed three rounds of grouping according to the integrated classification frameworks of GOLD 2015, GOLD 2025, and GOLD 2026, and further categorised patients based on migration from GOLD 2025 to GOLD 2026 into four subgroups: stable B (n = 220), shift-to-E (n = 242), stable E (n = 490), and others (n = 14). Among patients classified as group B under GOLD 2025 (n = 462), 242 (52.38%) were reclassified as group E under GOLD 2026 (**Figure 1**, Panel C), indicating substantial re-stratification associated with the updated framework.

**Figure 2:**
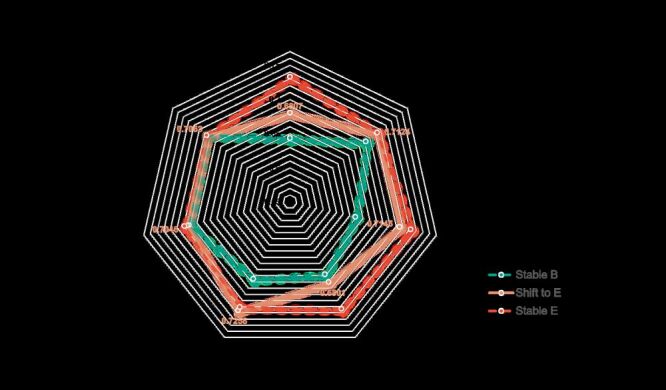
Radar Plot of E-Likeness Score Across GOLD 2026 Subgroups. The radar plot visualises the multidimensional clinical profile of the shift-to-E subgroup relative to the stable B and stable E subgroups across seven predefined domains. Within each domain, an E-likeness score was generated using a generalised linear model, with stable B and stable E as reference groups, assigned values of zero and one, respectively. Higher scores indicate greater resemblance to the stable E profile within that domain. These scores are intended as descriptive measures of multidomain similarity rather than domain-wise statistical comparisons. The variables included in each domain are listed in Table S3 in the **Online Supplementary Document**.

To characterise the phenotypic spectrum of the shift-to-E subgroup, we used a domain-based supervised similarity-scoring approach to assess its resemblance to stable B and stable E across seven domains (**Figure 2**; Table S3 in the **Online Supplementary Document**). Overall, shift-to-E showed intermediate patterns between stable B and stable E across these domains, indicating a B-E transitional phenotype. The radar plot indicated multi-domain similarity between the shift-to-E and stable E subgroups, especially in baseline vaccination, baseline symptoms, and inflammation levels, as reflected in the E-likeness score.

### Acute exacerbation risk during the follow-up period

**Figure 3:**
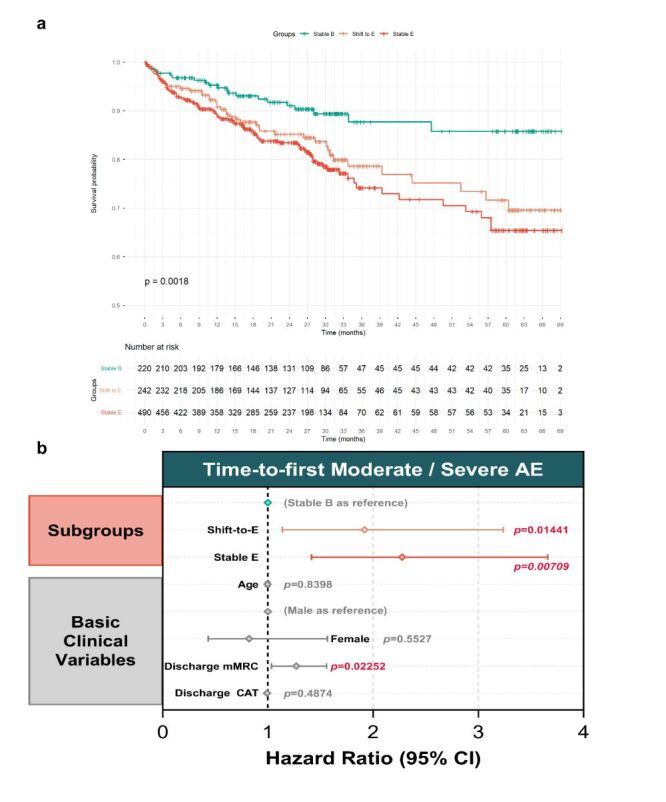
Risk of first moderate or severe acute exacerbation of COPD (AECOPD) during follow-up. Panel A. Kaplan-Meier curves for time to first moderate/severe AECOPD among GOLD subgroups. Panel B. Forest plot of hazard ratios for time to first moderate/severe AECOPD from Cox regression analysis.

During the average 10.3 months of follow-up after discharge, time to first moderate-to-severe acute exacerbation differed significantly across the three subgroups (log-rank *P* = 0.0018) (**Figure 3**, Panel A). Bonferroni-adjusted pairwise comparisons indicated that the overall difference was driven mainly by stable B *vs*. shift-to-E (adjusted *P* = 0.03292) and stable B *vs*. stable E (adjusted *P* = 0.001209). In contrast, shift-to-E and stable E did not observe a significant difference (adjusted *P* = 0.9760). These findings suggest that the event-free survival curve for shift-to-E was closer to that of stable E and substantially different from that of stable B.

Cox proportional hazards models further confirmed these differences. In univariable models, both shift-to-E and stable E showed significantly higher hazards of first moderate-to-severe exacerbation compared with stable B (HR = 1.94; 95% CI = 1.15–3.27, *P* = 0.01271 and HR = 2.31; 95% CI = 1.44–3.72, *P* = 0.00052). After adjustment for age, sex, discharge mMRC, and discharge CAT score, the associations remained significant (HR = 1.92; 95% CI = 1.14–3.23, *P* = 0.01441 and HR = 2.27; 95% CI = 1.41–3.66, *P* = 0.000709) (**Figure 3**, Panel B). The results above indicated a significantly elevated hazard in shift-to-E compared with stable B. The PH assumption was evaluated using Schoenfeld residual tests, and none of the covariates, including the global test, indicated a violation (*P* > 0.05). Sensitive analysis using Firth penalised Cox regression yielded consistent estimates (Table S4 and S5 in the **Online Supplementary Document**).

In addition, logistic regression using ‘≥1 severe exacerbation during follow-up’ as a binary endpoint showed increased odds for shift-to-E and stable E compared with stable B (odds ratio (OR) = 1.89; 95% CI = 1.24–2.91, *P* = 0.0032 and OR = 2.38; 95% CI = 1.63–3.50, *P* < 0.0001), suggesting that the burden of severe exacerbations in shift-to-E also resembled that of stable E, with the statistically significant difference compared with stable B.

### Symptom burden

Symptom burden analyses were conducted among patients with available follow-up symptom data (n = 850). Kruskal-Wallis tests indicated significant groupwise differences in discharge mMRC minus baseline mMRC score (ΔmMRC), follow-up CAT, follow-up mMRC, and follow-up discharge CAT score minus baseline CAT score (ΔCAT) (discharge ΔmMRC *P* = 0.00048; follow-up CAT *P* = 0.00033; follow-up mMRC *P* = 0.00104; follow-up ΔCAT *P* = 0.0329). Benjamini-Hochberg (BH)-adjusted pairwise Wilcoxon tests showed that differences in discharge ΔmMRC and follow-up ΔCAT were mainly driven by stable B *vs*. stable E, whereas no evidence of difference was observed between shift-to-E and stable B (*P* > 0.05); in contrast, follow-up CAT and mMRC demonstrated significant between-group differences (**Table 1**). After further adjustment for baseline symptom burden using analysis of covariance, follow-up CAT and mMRC remained significantly higher in shift-to-E (adjusted x̄ CAT = 14.83, adjusted x̄ mMRC = 2.191) and stable E (adjusted x̄ CAT = 15.30, adjusted x̄ mMRC = 0.214) than in stable B (adjusted x̄ CAT = 13.12, adjusted x̄ mMRC = 1.948). At the same time, no significant difference was observed between shift-to-E and stable E (Table S6 in the **Online Supplementary Document**). Overall, shift-to-E exhibited a heavier symptom burden than stable B and a profile close to stable E.

**Table 1:** BH-adjusted pairwise Wilcoxon test of symptom burden among subgroups*

Variable and group 1	Group 1	Group 2	N1	N2	Statistic	*P*-value
ΔCAT						
*Stable B*		Shift to E	220	242	29,329.5	0.088
*Stable B*		Stable E	220	490	59,547.5	0.076
*Shift to E*		Stable E	242	490	59,804.5	0.848
ΔmMRC						
*Stable B*		Shift to E	220	242	29,360.5	0.066
*Stable B*		Stable E	220	490	63,271.5	0.000309
*Shift to E*		Stable E	242	490	63,562.0	0.098
ΔVAS						
*Stable B*		Shift to E	220	242	26,676.0	0.969
*Stable B*		Stable E	220	490	55,341.5	0.897
*Shift to E*		Stable E	242	490	60,699.0	0.897
Follow-up CAT						
*Stable B*		Shift to E	199	220	18,627.0	0.013
*Stable B*		Stable E	199	431	34,460.5	0.000216
*Shift to E*		Stable E	220	431	44,927.0	0.274
Follow-up mMRC						
*Stable B*		Shift to E	199	220	19,081.5	0.02
*Stable B*		Stable E	199	431	35,648.0	0.000714
*Shift to E*		Stable E	220	431	45,439.0	0.347
Follow-up ΔCAT						
Stable B		Shift to E	199	220	19,493.0	0.079
*Stable B*		Stable E	199	431	37,471.0	0.032
*Shift to E*		Stable E	220	431	46,397.0	0.655
Follow-up ΔmMRC						
*Stable B*		Shift to E	199	220	19,392.5	0.066
*Stable B*		Stable E	199	431	38,792.0	0.066
*Shift to E*		Stable E	220	431	48,260.0	0.695

CAT – COPD assessment test, COPD – chronic obstructive pulmonary disease, mMRC – modified Medical Research Council dyspnea scale, VAS – visual analogue scale for dyspnea

*Adjusted *P*-value was calculated by the Benjamini-Hochberg procedure for post-hoc pairwise comparisons. Δ scores for CAT, mMRC, and VAS were defined as discharge scores minus baseline scores. Follow-up Δ scores for CAT, mMRC, and VAS were defined as the difference between follow-up and discharge scores.

### Care need

We evaluated care needs for home NIPPV and home oxygen therapy during follow-up using logistic regression, adjusting for age, sex, smoking index, baseline CAT, and baseline mMRC score. For home NIPPV, the ORs relative to stable B were 1.32 for shift-to-E and 1.64 for stable E, with no statistically significant difference. For home oxygen therapy, stable E had significantly higher odds than stable B, whereas shift-to-E showed a trend toward higher odds, though not statistically significant. Gamma regression analyses of treatment duration for home oxygen and home NIPPV therapy indicated a significant difference between stable B and stable E, but not between stable B and the shift-to-E subgroup (**Table 2**).

**Table 2:** Logistics and gamma regression of care needs among subgroups

Variables and subgroups	Adjusted x̄/adjusted probability (95% CI)*†	SE	Ratio/OR (95% CI)*†
Home NIPPV			
*Stable B*	0.09383 (0.06043–0.1429)*	0.2430	Ref.
*Shift to E*	0.120118 (0.08427–0.1684)*	0.2012	1.32 (0.72–2.45)*
*Stable E*	0.1451 (0.1148–0.1818)*	0.1375	1.64 (0.98–2.86)*
Home oxygen			
*Stable B*	0.3528 (0.2895–0.4217)*	0.1485	Ref.
*Shift to E*	0.4212 (0.3588–0.4861)*	0.1340	1.33 (0.91–1.97)*
*Stable E*	0.5104 (0.4642–0.5564)*	0.09441	1.91 (1.36–2.70)*
Home NIPPV hours			
*Stable B*	5.68 (4.01–8.06)**†**	0.17803	Ref.
*Shift to E*	7.38 (5.55–9.82)**†**	0.145436	1.30 (0.83–2.01)**†**
*Stable E*	7.06 (5.94–8.40)**†**	0.088445	1.24 (0.82–1.83)**†**
Home oxygen hours			
*Stable B*	9.38 (7.64–11.50)**†**	0.104497	Ref.
*Shift to E*	8.83 (7.40–10.54**†**	0.090143	0.94 (0.72–1.23)**†**
*Stable E*	8.32 (7.43–9.31)**†**	0.057558	0.89 (0.70–1.12)**†**

CI – confidence interval, NIPPV – non-invasive positive pressure ventilation, OR – odds ratio, ref – reference, SE – standard error, x̄ – mean

*Logistics regression.

†Gamma regression.

### Clinical features

Baseline hospital-stay lengths differed significantly across groups (Kruskal-Wallis *P* = 0.00229). BH-adjusted pairwise comparisons showed significant differences between stable B and shift-to-E (adjusted *P* = 0.043) and between stable B and stable E (adjusted *P* = 0.001). At the same time, no significant difference was observed between shift-to-E and stable E (adjusted *P* = 0.33). The results above indicate that higher demand for resource utilisation is associated with the higher-risk profile of shift-to-E.

In the age- and sex-adjusted gamma regression model, no significant differences in CRP, inpatient charges, or inpatient days were observed between subgroups. Kruskal-Wallis tests showed no significant difference between the shift-to-E and stable B subgroups; however, significant differences between the stable E and stable B subgroups were revealed in oxygen pressure and saturation (**Table 3**).

**Table 3:** Statistical analysis of clinical characteristics among subgroups

Variables and subgroups	Adjusted x̄ (95% CI)	SE	Ratio (95% CI)*	*P*-value†
CRP				
*Stable B*	31.80 (24.79–40.79)	0.127084	Ref.	
*Shift to E*	33.55 (26.47–42.51)	0.120867	1.06 (0.75–1.48)*	
*Stable E*	28.35 (23.87–33.66)	0.087623	0.89 (0.66–1.20)*	
Inpatient charge				
*Stable B*	16,406.5 (14,1306.0–19,049.0)	0.07619	Ref.	
*Shift to E*	14,792.8 (12,872.1–17,000.0)	0.07096	0.90 (0.74–1.10)*	
*Stable E*	14,997.0 (13,531.0–16,621.9)	0.05249	0.91 (0.76–1.09)*	
Inpatient days				
*Stable B*	8.24 (6.63–10.25)	0.110951	Ref.	
*Shift to E*	12.31 (10.01–15.13)	0.10539	1.49 (1.10–2.02)*	
*Stable E*	9.78 (8.45–11.31)	0.074371	1.18 (0.91–1.53)*	
pO_2_				
*Stable B*	82.42 (77.40–87.43)	2.554067		Ref.
*Shift to E*	78.26 (73.54–82.99)	2.40739		0.2305†
*Stable E*	76.13 (72.64–79.62)	1.777116		0.04053†
SO_2_				
*Stable B*	94.15 (93.27–95.02)	0.446974		Ref.
*Shift to E*	93.01 (92.18–93.84)	0.421305		0.06078†
*Stable E*	92.82 (92.20–93.42)	0.311004		0.01322†

CI – confidence interval, CRP – C-reactive protein, pO_2_ – arterial partial pressure of oxygen, SO2 – arterial blood oxygen saturation, ref – reference, SE – standard error, x̄ – mean

*Gamma regression.

†Kruskal-Wallis test.

In summary, the shift-to-E subgroup showed an overall transition from stable B to stable E across multiple domains. It more closely resembled stable E on selected key outcomes.

## DISCUSSION

This study is the first to quantify and characterise the shift-to-E patients identified by the updated GOLD criteria in a real-world cohort. Comprising 52.38% of the former group B, this subgroup showed a gradient phenotype spanning lung function, systemic inflammation, and baseline symptoms. Their risk of a first moderate-to-severe exacerbation differed significantly from stable B (adjusted HR = 1.92, *P* = 0.001441), with no significant difference between shift-to-E and stable E. Similarly, after adjustment, their symptom burden matched that of stable E and exceeded that of stable B. These findings suggested that the GOLD 2026 revision identifies a clinically meaningful moderate-to-high-risk subgroup. This resemblance should be interpreted as multidimensional and outcome-oriented rather than as complete equivalence across every individual clinical domain.

Although the lung-function sub-domain showed less visual separation than the others (**Figure 3**), this does not negate the overall interpretation of a B-to-E transitional phenotype, which may be rooted in underlying pathophysiological mechanisms. The elevated gradient of systemic inflammation in this subgroup, which falls between those of group B and group E, could suggest that a chronic, subclinical inflammatory state may serve as the ordinary soil underlying its more active disease course and increased susceptibility to acute exacerbations [14]. A documented moderate exacerbation may mark the transition of these patients from a relatively stable disease phase to a vulnerable, unstable state, which likely reflects their inherent ‘exacerbation-prone’ endotype characterised by local airway immune defence defects, microbiome dysbiosis, *etc.* [15,16]. Meanwhile, this subgroup may present both a heavier daily symptom burden and a higher risk of future acute exacerbations, suggesting that these two key clinical outcomes may share common upstream pathways such as oxidative stress, the impact of inadequately controlled comorbidities (*e.g.* cardiovascular diseases, anxiety, and depression), or persistent dynamic hyperinflation and respiratory mechanical load [17–19]. In-depth exploration of these underlying mechanisms will be crucial for understanding and intervening in this transitional phenotype in the future.

This study holds specific practical value in advancing precision management, optimising treatment decisions, and allocating resources. First, from a prognostic perspective, the revision of the GOLD 2026 threshold appears to improve risk stratification by identifying a previously underrecognised subgroup of patients with more adverse short-term outcomes within the persistently low-risk stable B subgroup [8]. These findings support closer follow-up and careful reassessment of patients at high risk of acute exacerbation after discharge. Second, this update enables the effective identification of occult high-risk patients. Under the previous criteria, these patients with only a single moderate exacerbation were classified into group B and may not have received adequate attention [20,21]. The updated criteria and their validation results suggest that they constitute a potentially high-risk population requiring enhanced monitoring and proactive management, thereby facilitating early intervention and precise stratification in COPD management [22,23]. Our findings are therefore consistent with the rationale underlying the GOLD 2026 update, which emphasises that even a single moderate exacerbation may indicate an increased future risk [9,24,25].

The main strengths of this study lie in its prospective, multi-centre cohort design, which ensures standardised collection of high-quality data and effectively reduces the common biases associated with retrospective studies. By conducting dynamic ‘head-to-head’ comparisons of the three GOLD criteria in the same patient population, the study clearly and intuitively reveals the specific clinical impacts and population reclassification resulting from the evolution of the classification criteria. In addition, the study adopted a comprehensive, multidimensional outcome assessment framework that not only focuses on acute exacerbation as a hard endpoint but also evaluates patients’ symptom burden, quality of life, and health care resource utilisation, thereby depicting the overall disease burden in three dimensions.

However, this study also has several limitations. First, the study cohort was derived in Hunan Province, China, and local population characteristics, environmental factors, and medical practices may influence its results. The generalisability of the findings to other regions worldwide remains to be validated across diverse ethnic groups and health care systems. In addition, the cohort was predominantly older and male, which may further limit extrapolation to female patients and populations with different demographic structures. Baseline disease duration differed across subgroups and was not included in the primary adjustment models, so residual confounding cannot be excluded. Second, the average follow-up duration of approximately 10.3 months is sufficient to assess short- to medium-term risk of acute exacerbation. Still, it is not long enough to evaluate the impact of the updated criteria on ultimate disease-course-related outcomes, such as all-cause mortality [26]. We also did not evaluate more granular treatment-related data, such as oxygen flow rate, ventilator settings, or baseline maintenance pharmacotherapy patterns, which may have contributed to heterogeneity in outcomes. In addition, analysis-specific missingness may lead to differing effective sample sizes across endpoints, particularly in symptom-burden analyses, potentially introducing selection bias. Finally, this study primarily focuses on the characterisation and validation of clinical phenotypes, and the exploration of the underlying biological mechanisms driving the ‘shift-to-E’ phenotype, including specific inflammatory pathways and actionable biomarkers, remains preliminary.

## CONCLUSIONS

This prospective study validates the clinical value of the GOLD 2026 criteria in lowering the threshold for defining group E. The updated criteria reclassify more than half of the patients previously categorised as group B under GOLD 2025 into group E, forming a unique ‘shift-to-E’ subgroup. This subgroup exhibits transitional clinical features between group B and group E, with acute exacerbation risk, symptom burden, and health care needs similar to those of the stable group E but significantly higher than those of the stable group B. Despite limitations such as a single study population and a limited follow-up period, this study provides early real-world evidence for the clinical application of GOLD 2026, supporting the advancement of precision management of COPD.

**Data availability:** The datasets used in this investigation are available upon reasonable request from the corresponding author.

## Additional material


Online Supplementary Document
