# Supplementary material for: The high-risk B-to-E transitional phenotype: a prospective multi-centre validation of patient reclassification under the updated GOLD 2026 criteria
Source: J Glob Health. 2026 Jul 17;16:04205. doi: 10.7189/jogh.16.04205 (PMC13377587; doi:10.7189/jogh.16.04205)
Supplement: Online Supplementary Document [file jogh-16-04205-s001.pdf]

Supplement to: Zhang B, Zeng J, Zeng H. The high-risk B-to-E transitional phenotype: a prospective multi-centre validation of patient reclassification under the updated GOLD 2026 Criteria. J Glob Health. 2026;16:04205.

The High-Risk B-to-E Transitional Phenotype: A Prospective Multi-Center Validation of Patient Reclassification Under the Updated GOLD 2026 Criteria

Boyan Zhang [1#], Junhao Zeng [2#], Huihui Zeng [1], Zhongshang Dai [3], Yan Chen [1]*

[1] Department of Respiratory and Critical Care Medicine, The Second Xiangya Hospital of Central South University, Changsha, China

[2] National Clinical Research Center for Endocrine and Metabolic Diseases and Department of Metabolism and Endocrinology, The Second Xiangya Hospital of Central South University, Changsha, China

[3] Department of Infectious Diseases, The Second Xiangya Hospital, Central South University, Changsha, China

[#] Boyan Zhang and Junhao Zeng contributed equally to this article.

[*] Correspondence to Zhongshang Dai (Email: dzs8240@csu.edu.cn)

Supplementary materials

Table S1 The Strengthening the Reporting of Observational Studies in Epidemiology (STROBE) Statement of this study.

Table S2 Baseline Characteristics Stratified by Subgroups.

Table S3 7 Subdomains of Baseline Information.

Table S4 ZPH Test Result for Cox Regression Model.

Table S5 Firth Penalized Cox Proportional Risk Regression and Pairwise Analysis.

Table S6 ANCOVA After BH Adjustment of Symptoms Burden Among Subgroups.

Table S1 The Strengthening the Reporting of Observational Studies in Epidemiology (STROBE) Statement of this study.

	Item No	Recommendation	Page No
**Title and abstract**	1	(*a*) Indicate the study's design with a commonly used term in the title or the abstract	1
		(*b*) Provide in the abstract an informative and balanced summary of what was done and what was found	1
**Introduction**			
Background/rationale	2	Explain the scientific background and rationale for the investigation being reported	1
Objectives	3	State specific objectives, including any prespecified hypotheses	1-2
**Methods**			
Study design	4	Present key elements of study design early in the paper	2
Setting	5	Describe the setting, locations, and relevant dates, including periods of recruitment, exposure, follow-up, and data collection	2
Participants	6	(*a*) Give the eligibility criteria, and the sources and methods of selection of participants. Describe methods of follow-up	2
		(*b*) For matched studies, give matching criteria and number of exposed and unexposed	N/A
Variables	7	Clearly define all outcomes, exposures, predictors, potential confounders, and effect modifiers. Give diagnostic criteria, if applicable	2-3
Data sources/ measurement	8*	For each variable of interest, give sources of data and details of methods of assessment (measurement). Describe comparability of assessment methods if there is more than one group	2-3
Bias	9	Describe any efforts to address potential sources of bias	3
Study size	10	Explain how the study size was arrived at	2
Quantitative variables	11	Explain how quantitative variables were handled in the analyses. If applicable, describe which groupings were chosen and why	3
Statistical methods	12	(*a*) Describe all statistical methods, including those used to control for confounding	3
		(*b*) Describe any methods used to examine subgroups and interactions	3
		(*c*) Explain how missing data were addressed	3
		(*d*) If applicable, explain how loss to follow-up was addressed	2
		(*e*) Describe any sensitivity analyses	3
**Results**			
Participants	13*	(a) Report numbers of individuals at each stage of study—eg numbers potentially eligible, examined for eligibility, confirmed eligible, included in the study, completing follow-up, and analysed	4
		(b) Give reasons for non-participation at each stage	4
		(c) Consider use of a flow diagram	4
Descriptive data	14*	(a) Give characteristics of study participants (eg demographic, clinical, social) and information on exposures and potential confounders	4
		(b) Indicate number of participants with missing data for each variable of interest	4
		(c) Summarise follow-up time (eg, average and total amount)	4
Outcome data	15*	Report numbers of outcome events or summary measures over time	4-5

Main results	16	(*a*) Give unadjusted estimates and, if applicable, confounder-adjusted estimates and their precision (eg, 95% confidence interval). Make clear which confounders were adjusted for and why they were included	5
		(*b*) Report category boundaries when continuous variables were categorized	3
		(*c*) If relevant, consider translating estimates of relative risk into absolute risk for a meaningful time period	5
Other analyses	17	Report other analyses done—eg analyses of subgroups and interactions, and sensitivity analyses	5-7
**Discussion**			
Key results	18	Summarise key results with reference to study objectives	7
Limitations	19	Discuss limitations of the study, taking into account sources of potential bias or imprecision. Discuss both direction and magnitude of any potential bias	8
Interpretation	20	Give a cautious overall interpretation of results considering objectives, limitations, multiplicity of analyses, results from similar studies, and other relevant evidence	7-8
Generalisability	21	Discuss the generalisability (external validity) of the study results	8
**Other information**			
Funding	22	Give the source of funding and the role of the funders for the present study and, if applicable, for the original study on which the present article is based	9

*Give information separately for exposed and unexposed groups.

Note: An Explanation and Elaboration article discusses each checklist item and gives methodological background and published examples of transparent reporting. The STROBE checklist is best used in conjunction with this article (freely available on the Web sites of PLoS Medicine at http://www.plosmedicine.org/, Annals of Internal Medicine at http://www.annals.org/, and Epidemiology at http://www.epidem.com/). Information on the STROBE Initiative is available at http://www.strobe-statement.org.

Reference

von Elm E, Altman DG, Egger M, Pocock SJ, Gøtzsche PC, Vandenbroucke JP, et al. The Strengthening the Reporting of Observational Studies in Epidemiology (STROBE) statement: guidelines for reporting observational studies. Lancet. 2007;370:1453–7. Medline:18064739 doi:10.1016/S0140-6736(07)61602-X

Table S2 Baseline Characteristics Stratified by Subgroups.

Variable	Overall (N=966)	Stable B (N=220)	Shift-to-E (N=242)	Stable E (N=490)	Others (N=14)	*P* value
Age (years)	70.00 (64.00, 75.00)	70.00 (62.00, 75.00)	69.00 (63.00, 75.00)	70.00 (65.00, 74.00)	66.50 (59.25, 75.00)	0.8566
BMI (kg/m^2)	20.96 (18.47, 23.88)	22.08 (19.86, 24.63)	21.03 (18.80, 23.98)	20.71 (17.92, 23.44)	21.60 (18.12, 25.37)	0.0001726
Sex						0.3519
Male	894 (92.5%)	199 (90.5%)	223 (92.1%)	458 (93.5%)	14 (100.0%)	
Female	72 (7.5%)	21 (9.5%)	19 (7.9%)	32 (6.5%)	0 (0.0%)	
Education						0.005999
Primary or Less	478 (49.5%)	114 (51.8%)	113 (46.7%)	248 (50.6%)	3 (21.4%)	
Middle School	249 (25.8%)	51 (23.2%)	70 (28.9%)	125 (25.5%)	3 (21.4%)	
High School/Technical Secondary School	167 (17.3%)	38 (17.3%)	50 (20.7%)	72 (14.7%)	7 (50.0%)	
University/College or More	72 (7.5%)	17 (7.7%)	9 (3.7%)	45 (9.2%)	1 (7.1%)	
Smoking						0.179
Never	145 (15.0%)	37 (16.8%)	41 (16.9%)	67 (13.7%)	0 (0.0%)	
Current	209 (21.6%)	56 (25.5%)	53 (21.9%)	96 (19.6%)	4 (28.6%)	
Previous	612 (63.4%)	127 (57.7%)	148 (61.2%)	327 (66.7%)	10 (71.4%)	
Smoking index	30.00 (0.00, 50.00)	20.00 (0.00, 50.00)	20.00 (0.00, 40.00)	30.00 (0.00, 50.00)	26.50 (1.88, 47.50)	0.05792
Baseline CAT score	21.00 (16.00, 24.00)	19.00 (15.00, 22.25)	20.00 (16.00, 24.00)	22.00 (17.00, 25.00)	5.00 (4.25, 6.00)	<0.0001
Baseline VAS score	7.00 (6.00, 8.00)	7.00 (5.00, 8.00)	7.00 (5.00, 8.00)	7.00 (6.00, 8.00)	3.50 (1.50, 7.00)	0.002046
In-hospital days	8.00 (6.00, 11.00)	7.00 (6.00, 10.00)	8.00 (6.00, 11.00)	8.00 (7.00, 11.00)	7.50 (5.00, 10.75)	0.0059

						44
In-hospital charge	11394.10 (8683.29, 16174.80)	11166.86 (8054.65, 15059.26)	11406.01 (8893.19, 16788.90)	11259.00 (8888.50, 16450.69)	12590.00 (9147.21, 14538.59)	0.416
pO_2 (mmHg)	73.00 (63.00, 85.80)	76.00 (65.00, 86.75)	71.15 (61.95, 85.62)	71.00 (62.00, 85.00)	82.50 (76.50, 88.75)	0.02659
SO_2 (%)	95.00 (92.00, 96.50)	95.00 (93.00, 97.00)	94.00 (91.00, 96.00)	94.00 (92.00, 96.00)	96.00 (95.00, 97.00)	0.01069
CRP (mg/L)	9.62 (3.63, 36.20)	11.05 (3.85, 40.47)	10.80 (4.10, 34.90)	8.66 (3.21, 36.05)	7.17 (4.08, 24.38)	0.4099
FEV_1 (L)	0.81 (0.60, 1.17)	0.90 (0.67, 1.25)	0.83 (0.60, 1.17)	0.74 (0.57, 1.10)	1.53 (1.19, 1.98)	0.0108
FEV_1%pred	33.70 (24.40, 46.80)	36.70 (29.13, 48.98)	33.70 (23.85, 49.55)	30.80 (23.52, 42.65)	54.00 (46.30, 57.10)	0.004088
FEV_1 after bronchodilator (L)	0.85 (0.63, 1.26)	0.94 (0.69, 1.38)	0.89 (0.61, 1.29)	0.78 (0.60, 1.11)	1.78 (1.54, 1.94)	0.002321
$FEV_1\%_{pred}$ after bronchodilator	35.60 (24.70, 51.02)	40.15 (30.52, 53.75)	35.20 (24.20, 55.05)	33.60 (24.00, 46.70)	57.15 (54.73, 60.93)	0.005381
FEV_1/FVC	35.41 (25.50, 47.85)	41.09 (30.77, 54.15)	36.48 (27.34, 49.79)	32.70 (22.59, 43.22)	44.28 (40.76, 58.58)	0.001968
$FEV_1/FVC\%_{pred}$	48.60 (38.80, 64.50)	57.20 (45.58, 70.65)	51.95 (39.78, 66.07)	44.90 (36.10, 59.08)	57.90 (54.60, 77.80)	<0.0001
FEV_1/FVC after bronchodilator	0.38 (0.31, 0.51)	0.42 (0.36, 0.55)	0.40 (0.30, 0.51)	0.35 (0.29, 0.46)	0.51 (0.45, 0.59)	<0.0001
$FEV_1/FVC\%_{pred}$ after bronchodilator	48.90 (38.98, 63.65)	55.00 (46.50, 70.00)	51.05 (38.55, 64.80)	45.00 (36.50, 59.00)	67.20 (59.47, 77.17)	0.0002271
Years after COPD first diagnosis	5.00 (0.79, 10.00)	2.25 (0.00, 6.00)	4.00 (0.50, 9.00)	5.75 (3.00, 10.00)	0.65 (0.00, 2.75)	<0.0001
Baseline mMRC						0.0002

Dyspnea only with strenuous exercise	14 (1.4%)	5 (2.3%)	1 (0.4%)	4 (0.8%)	4 (28.6%)	
Dyspnea when hurrying or walking up a slight hill	102 (10.6%)	32 (14.5%)	22 (9.1%)	38 (7.8%)	10 (71.4%)	
Walks slower than people of the same age because of dyspnea or has to stop for breath when walking at own pace	265 (27.4%)	72 (32.7%)	84 (34.7%)	109 (22.2%)	0 (0.0%)	
Stops for breath after walking 100 yards (91 m) or after a few minutes	359 (37.2%)	80 (36.4%)	87 (36.0%)	192 (39.2%)	0 (0.0%)	
Too dyspneic to leave house or breathless when dressing	226 (23.4%)	31 (14.1%)	48 (19.8%)	147 (30.0%)	0 (0.0%)	
Influenza vaccination						0.7632
No	831 (86.0%)	191 (86.8%)	210 (86.8%)	417 (85.1%)	13 (92.9%)	
Yes	135 (14.0%)	29 (13.2%)	32 (13.2%)	73 (14.9%)	1 (7.1%)	
Pneumonia vaccination						0.4937
No	860 (89.0%)	200 (90.9%)	218 (90.1%)	429 (87.6%)	13 (92.9%)	
Yes	106 (11.0%)	20 (9.1%)	24 (9.9%)	61 (12.4%)	1 (7.1%)	
COVID-19 vaccination						0.008398
No	321 (33.2%)	79 (35.9%)	60 (24.8%)	179 (36.5%)	3 (21.4%)	
Yes	645 (66.8%)	141 (64.1%)	182 (75.2%)	311 (63.5%)	11 (78.6%)	
Emergency admitted						0.4879

No	809 (83.7%)	181 (82.3%)	197 (81.4%)	419 (85.5%)	12 (85.7%)	
Yes	157 (16.3%)	39 (17.7%)	45 (18.6%)	71 (14.5%)	2 (14.3%)	
Cough						0.22
No	116 (12.0%)	26 (11.8%)	25 (10.3%)	61 (12.4%)	4 (28.6%)	
Occasionally	664 (68.7%)	152 (69.1%)	176 (72.7%)	326 (66.5%)	10 (71.4%)	
Always	165 (17.1%)	38 (17.3%)	39 (16.1%)	88 (18.0%)	0 (0.0%)	
Continuous	21 (2.2%)	4 (1.8%)	2 (0.8%)	15 (3.1%)	0 (0.0%)	
Sputum						0.3043
No	104 (10.8%)	21 (9.5%)	27 (11.2%)	53 (10.8%)	3 (21.4%)	
Mild (10-50 mL)	612 (63.4%)	141 (64.1%)	163 (67.4%)	297 (60.6%)	11 (78.6%)	
Moderate (50-100 mL)	211 (21.8%)	49 (22.3%)	43 (17.8%)	119 (24.3%)	0 (0.0%)	
Severe (>100 mL)	39 (4.0%)	9 (4.1%)	9 (3.7%)	21 (4.3%)	0 (0.0%)	
Purulent sputum						0.1076
No	646 (66.9%)	151 (68.6%)	173 (71.5%)	311 (63.5%)	11 (78.6%)	
Yes	320 (33.1%)	69 (31.4%)	69 (28.5%)	179 (36.5%)	3 (21.4%)	
Wheezing						0.0003999
No	320 (33.1%)	77 (35.0%)	102 (42.1%)	133 (27.1%)	8 (57.1%)	
Yes	646 (66.9%)	143 (65.0%)	140 (57.9%)	357 (72.9%)	6 (42.9%)	
Running nose						0.4935
No	846 (87.6%)	194 (88.2%)	217 (89.7%)	422 (86.1%)	13 (92.9%)	
Yes	120 (12.4%)	26 (11.8%)	25 (10.3%)	68 (13.9%)	1 (7.1%)	
Fever						0.2873
No	847 (87.7%)	192 (87.3%)	215 (88.8%)	430 (87.8%)	10 (71.4%)	
Yes	119 (12.3%)	28 (12.7%)	27 (11.2%)	60 (12.2%)	4 (28.6%)	
Sore throat						0.8076

No	909 (94.1%)	208 (94.5%)	228 (94.2%)	459 (93.7%)	14 (100.0%)	
Yes	57 (5.9%)	12 (5.5%)	14 (5.8%)	31 (6.3%)	0 (0.0%)	
RICU admitted						0.1454
No	692 (93.4%)	169 (96.0%)	178 (91.8%)	336 (93.3%)	9 (81.8%)	
Yes	49 (6.6%)	7 (4.0%)	16 (8.2%)	24 (6.7%)	2 (18.2%)	
Oxygen therapy						0.1404
No	296 (37.4%)	57 (30.6%)	82 (39.4%)	151 (39.2%)	6 (50.0%)	
Yes	495 (62.6%)	129 (69.4%)	126 (60.6%)	234 (60.8%)	6 (50.0%)	
NIMV therapy						0.3449
No	649 (82.0%)	155 (83.3%)	162 (77.9%)	322 (83.6%)	10 (83.3%)	
Yes	142 (18.0%)	31 (16.7%)	46 (22.1%)	63 (16.4%)	2 (16.7%)	
EPAP						0.7041
No	770 (97.3%)	183 (98.4%)	202 (97.1%)	373 (96.9%)	12 (100.0%)	
Yes	21 (2.7%)	3 (1.6%)	6 (2.9%)	12 (3.1%)	0 (0.0%)	
Diabetes Mellitus						0.4477
No	100 (61.7%)	27 (71.1%)	23 (59.0%)	49 (59.8%)	1 (33.3%)	
Yes	62 (38.3%)	11 (28.9%)	16 (41.0%)	33 (40.2%)	2 (66.7%)	
Hyperlipidemia						0.1204
No	215 (55.4%)	41 (46.1%)	60 (57.7%)	113 (59.2%)	1 (25.0%)	
Yes	173 (44.6%)	48 (53.9%)	44 (42.3%)	78 (40.8%)	3 (75.0%)	

Table S3 7 Subdomains of Baseline Information.

Subdomains	Variables
Demographics	Gender, Age, BMI, Education level, Smoking, Smoking index
Baseline Vaccination	Influenza, Pneumonia, COVID-19
Baseline Symptoms	Baseline CAT score, Baseline mMRC score, Whether it is an emergency admission, Cough, Sputum, Purulent sputum, Running nose, Wheezing, Fever, Sore throat
Vital Signs	Temperature, Heart rate, Systolic blood pressure, Diastolic blood pressure, Respiratory rate
Inflammation Situation	WBC count, Neutrophil count, Eosinophil count, CRP, Procalcitonin
Lung Function	$FEV_1\%_{pred}$, FEV_1/FVC
Discharge Status	Discharge CAT, Discharge mMRC

BMI: Body Mass Index, CAT: COPD Assessment Test, mMRC: modified Medical Research Council dyspnea scale, WBC: White Blood Cell, CRP: C-Reactive Protein, $FEV_1\%_{pred}$: Forced Expiratory Volume in One second as a percentage of the predicted value; FEV_1/FVC: the ratio of forced expiratory volume in one second and forced vital capacity.

Table S4 ZPH Test Result for Cox Regression Model.

Term	χ^2	df	*P* Value
Group	0.803205	2	*0.669247*
Age	0.155841	1	*0.693015*
Sex	0.477557	1	*0.489531*
Discharge mMRC	2.182985	1	*0.139544*
Discharge CAT	0.071512	1	*0.789147*
GLOBAL	3.896133	6	*0.69073*

Table S5 Firth Penalized Cox Proportional Risk Regression and Pairwise Analysis.

Table S5a Firth Penalized Cox Proportional Risk Regression Model Analysis

	**Coeffcience**	**SE**	**Exp (95% CI)**	**χ^2**	***P* Value**
Shift to E	0.6470	0.2649	1.91 (1.15-3.26)	6.3787	*0.0115*
Stable E	0.8220081	0.2406784	2.28 (1.45-3.79)	13.77447	*0.000206118*

Table S5b Pairwise Analysis of the Firth Penalized Cox Regression Model

	**Shift to E**	**Stable E**
**Shift to E**	0.07018089	0.04698005
**Stable E**	0.04698005	0.05792611

Table S6 ANCOVA After BH Adjustment of Symptoms Burden Among Subgroups.

**Variable**	**Group Contrast**	**SE**	**t**	**Adjusted *P***
Follow-up	Stable B vs. Shift-to E	0.6754	2.540	0.01691
CAT	Stable B vs. Stable E	0.5934	3.669	0.000777
	Shift-to-E vs. Stable E	0.5736	0.8048	0.4212
Follow-up	Stable B vs. Shift-to E	0.08708	2.7898	0.00809
mMRC	Stable B vs. Stable E	0.07655	3.4706	0.001637
	Shift-to-E vs. Stable E	0.07400	0.3075	0.7586

The adjusted *P* value was calculated using the Bonferroni method across three subgroups.
